# Innovative formulation of microencapsulated “spirubiotic sachets” as a synbiotic treatment for metabolic syndrome patients

**DOI:** 10.1038/s41598-025-29579-x

**Published:** 2025-12-09

**Authors:** Moetazza M. Alshafei, Yasmin M. Ziada, Maha M. Saber, Eitedal M. Dawood, Ahmed M. Mabrouk, Tamer M. El-Messery, Fatma El-Zahraa Sayed M. Abdel-Fattah, Dina Mostafa Mohammed

**Affiliations:** 1 Nutrition and Food Sciences Department, National Research Centre, Dokki, Giza, 12622 Egypt; 2 Department of Complementary Medicine, National Research Centre, Giza, 12622 Egypt; 3 Dairy Department, National Research Centre, Dokki, Giza, 12622 Egypt

**Keywords:** Probiotic bacteria, Type 2 diabetes, Metabolic syndrome patients, Obesity, Spirulina platensis, Spirubiotic sachets, Biochemistry, Biotechnology, Diseases, Health care

## Abstract

*Spirulina platensis* (Sp) and probiotic bacteria (Pb) have been shown to have beneficial effects in controlling metabolic syndrome (MeS), characterized by the co-occurrence of obesity, dyslipidemia, hypertension, and type 2 diabetes. The objective of this research was to formulate an innovative microencapsulated powder consisting of a mixture of Sp and Pb, referred to as a “spirubiotic sachet,” and investigate its potential positive impact in managing hyperlipidemia, weight gain, hyperglycemia, and related problems in individuals with metabolic syndrome. Microencapsulated Sp with whey protein, flavored with a coconut taste, was obtained by spray drying, and HPLC analysis was performed. Probiotic bacteria were microencapsulated by freeze-drying, and the characterization of both microcapsules was done. The spirubiotic sachets contained both Sp and Pb. Physico-sensory tests were reported. Fifty diagnosed MeS cases were enrolled in this study. Pre- and post-treatment assessments of clinical, dietary, anthropometric data, and body composition measurements were conducted. Enzymatic colorimetric methods and ELISA kits were used to assess biochemical parameters. Patients received one spirubiotic sachet/day and a tailored diet regimen for 3 months. Administering a spirubiotic sachet to MeS patients effectively reversed the majority of metabolic abnormalities, bringing them closer to normal levels with decreased dietary calories, improved anthropometric measures, altered body composition, and normalized biochemical parameters. In conclusion, Sp and Pb work together synergistically to help manage people with MeS and related diseases. Spirubiotic sachets can be innovative with beneficial health effects.

## Introduction

 Metabolic syndrome (MeS) is a significant health issue characterized by the simultaneous occurrence of three or more metabolic diseases, including central obesity, dyslipidemia, hypertension, hyperglycemia, and cardiovascular disease. In the realm of public health, the issue has come to light as a significant one, and its prevalence is likely to increase in the future^1^. Obesity and diabetes are inextricably linked to the development of chronic, low-grade systemic inflammation, which, in turn, drives various inflammatory diseases. Ensuring that estrogen levels in females are within the normal range is crucial in preventing the accumulation of fat in essential organs, hence reducing the high occurrence of cardiovascular disease in females. The prior trait of normal fat deposition beyond essential organs will not be present in females impacted by MeS^2^.

Probiotic bacteria (Pb) provide several health advantages^3,4^. It decreases the activity of mutagenic enzymes^5^. Additionally, it improves cholesterol and sugar levels in individuals with type 2 diabetes and liver steatosis, as well as in rats with metabolic syndrome (MeS). According to other studies, an imbalance of gut microbiota may lead to reproductive issues, including polycystic ovarian syndrome and aberrant estrogen levels^3,6^. This imbalance also contributes to metabolic disturbances in blood cholesterol and glucose levels, as revealed by Hanafi et al.^2^.

*Spirulina platensis* (Sp) is a blue-green algae that thrives in both fresh and saltwater environments. It is known for its high protein content, which may reach up to 70%. Additionally, *Spirulina platensis* contains a variety of beneficial compounds, including vitamins, β-carotenes, minerals, phenolic acids, tocopherols, and γ-linolenic acid. Several toxicological tests have confirmed its biosafety. Moreover, the use of *Spirulina platensis* has been associated with a reduced risk of cardiovascular events^3,7^. As a prebiotic, *Spirulina platensis* can release extracellular carbohydrates (fructooligosaccharides) and other growth substances that may promote the growth of probiotic bacteria in the gut. This symbiotic relationship will enhance the health benefits and lipid pathways of both the bacteria and the human body^2,3,5^. For decades, prebiotic polysaccharides derived from algae, including fucans, laminarins, and alginates, have been used to improve both human and animal health^8^.

By regulating obesity, diabetes, and related illnesses, nutrients such as probiotic bacteria and *Spirulina platensis* microalgae contain physiologically active substances that may improve metabolic state^3,4^. In the earlier animal investigation using the MeS rat model, we observed that Sp and Pb could reduce levels of serum cholesterol, LDL, triglycerides, and blood glucose^3^. Spirulina has shown beneficial effects on the female reproductive system, which may trigger MeS^2^. Similar findings were found in several human trials^6,9^.

Microencapsulation is a technique of enveloping minuscule particles or droplets with a protective layer, forming small capsules. Microencapsulation is frequently achieved through spray drying and freeze drying, which are widely employed methods. Spray drying offers numerous benefits for microencapsulation, including the ability to generate homogeneous particles and achieve high encapsulation efficiency^10^. Microencapsulation protects active substances from external damage, delivers them unchanged to targeted organs, and controls the release of active substances^8,11^. The commonly employed coating materials, such as proteins (e.g., casein and whey protein), can form encapsulated materials. Whey protein, as an encapsulation wall, is an additive that exhibits excellent emulsifying and film-forming properties^12^, in addition to its beneficial health effects that can aid in controlling obesity and enhancing muscle mass growth^10^.

The central issue in developing a combined therapeutic for metabolic syndrome (MeS) is ensuring the stable and effective synergy of its known beneficial components: Spirulina (Sp) and probiotic bacteria (Pb). While both Sp and Pb are individually effective against MeS symptoms (such as obesity and dyslipidemia), mixing them as powders leads to poor probiotic survival due to Pb’s sensitivity to environmental factors, including temperature, moisture, and stomach acid, resulting in inconsistent or diminished therapeutic effects. This creates a knowledge gap concerning a stable, consumer-friendly “spirubiotic” formulation that maintains the viability and function of both components. Therefore, this study was primarily designed to develop a novel synbiotic microencapsulated powder, designated a “spirubiotic sachet,” comprising a precise mixture of Sp (likely *Spirulina platensis*, serving as a prebiotic/nutraceutical component) and Pb (likely a specific probiotic bacterium). For enhanced stability and palatability, the microcapsules utilized whey protein concentrate—a food-grade protein polymer—as the encapsulating matrix, with the addition of coconut flavoring. A secondary objective of the research was to conduct a rigorous investigation into the therapeutic efficacy of administering these microencapsulated sachets for the comprehensive management of interconnected metabolic syndrome pathologies, specifically focusing on their potential to ameliorate hyperlipidemia (dyslipidemia), mitigate excessive weight gain (obesity), and improve systemic hyperglycemia.

## Materials and methods

### Materials

Whey Protein Concentrate–Coconut (WPCC) (80% Protein) was purchased from Decathlon Co., France. Sp was purchased from a unit of algae production in NRC. The Lactobacilli strains (Pb) were acquired from the Northern Regional Research Laboratory (NRRL) (Peoria, Illinois, USA). The kits for spectrophotometry used in the quantitative measurement were procured from the Biodiagnostic Company of the Egyptian Company for Biotechnology (Giza, Egypt). The ELISA kits were obtained from Sunlong Biotech (Hangzhou City, Zhejiang Province, China).

### Methods

####  Microencapsulation of *Spirulina platensis* (Sp)

##### Preparation of spirulina microcapsules (SMs)

To create spirulina microcapsules, the first procedure involves dissolving WPCC in distilled water (dw) at a concentration of 10% w/v. This mixture becomes the substance from which the microcapsules’ walls will be made. Subsequently, the WPCC solution was supplemented with spirulina, the fundamental component. The homogenization process was completed by employing a high-shear mixer—more precisely, an Ultra-Turrax mixer—that ran at 4,000 rpm for 10 min. The ratio of spirulina to the WPCC was 1:10 (W/W). With a 10-minute break in between, the procedure was split into two 5-minute halves. To address dehydration, the recipe was subjected to a spray drying process utilizing a Buchi Mini Spray Dryer B-290 from Switzerland, resulting in the production of powdered substances. The inlet and output air temperatures for the spray drying process were 140 ± 5 °C (with an atomizing air flow of 5 cm³/min) and 70 ± 5 °C (with an airflow of 0.67 m³/min), respectively. The powder obtained consisted of 1 g of row Sp for every 10 g of SMs.

##### Characterization of spirulina microcapsules (SMs)

*Determination of zeta potential and particle size distribution*: Using a dynamic light scattering device (Nano ZS, Malvern Instruments, Worcestershire, UK), the zeta potential and particle size measurements were carried out. Lecithin was found to have a refractive index of 1.37 ± 0.02. A concentration of 0.1% (w/w) was achieved by diluting the SMs solutions before the measurements.

*Surface morphology analysis of SMs powder*: Field-emission scanning electron microscopes (FE-SEM, FEI Quanta FEG 250) were used to analyze the morphology and structure of the spray-dried samples. A broad-area low-vacuum secondary electron detector (LFD) 2, operating at a potential of 2.00 kV and a pressure of 70 pascals, was used for imaging, and it was not coated with any compounds that might improve conductivity.

*Determination of total phenolic content (TPC of SMs powder)*: The Folin-Ciocalteu technique was used to ascertain the TPC. In a mixture of ethanol, acetic acid, and purified water (48:2:50, v/v/v), the SMs powder (SMsp) was dissolved at a ratio of 1:10 (w/v). A total of 200 µl of dissolved SMsp and 1.58 ml of clean water were combined, and then 100 µl of Folin-Ciocalteu reagent (1:10, v/v) was added. Following an 8-minute incubation period at 25 °C in the absence of light, 300 µL of a 20% sodium carbonate solution was introduced. The samples were vortexed, and after 30 min of dark incubation at 40 °C, the UV-V spectrophotometer (BioTek Instruments, Winooski, USA) was used to detect the absorbance at 765 nm. The Total Polyphenol Content (TPC) was quantified in mg of gallic acid (GA)/g of sample, based on dry weight, using a calibration curve. The calibration curve had an R2 value greater than 0.99^13^.

*Determination of free phenolic content (FPC)*: To identify the amount of phenolic content on the surface of the SMs powder, it was dissolved in a solution of 50% ethanol at a ratio of 1:10 (weight/volume). The surface phenolic content was evaluated and measured using the same procedure described in the section on total phenolic content^14^.

*Calculation of encapsulation efficienc*y: In terms of TPC and FPC, which were previously assessed, the content and position of SMsp phenolics were measured to ascertain the encapsulation effectiveness of SMs. The calculation of the encapsulation efficiency was determined using Eq. (1)^14^.

1 $$\:EE\%=\frac{\:\text{T}\text{P}\text{C}-\:\text{F}\text{P}\text{C}}{\text{T}\text{P}\text{C}}x\:100$$

Where: TPC: Total phenolic compounds (encapsulated + non-encapsulated).

FPC: Free phenolic compounds in the supernatant (non-encapsulated).

Determination of antioxidant activity of *SMs*: The DPPH test was used to evaluate antioxidant activity. The antioxidant activity was quantified by measuring the amount of Trolox equivalents (µmol/g) in the DPPH tests^15^.

*HPLC conditions of SMs*: An HPLC analysis was performed using Agilent 1260 series equipment, with separation achieved on an Eclipse C18 column (4.6 mm diameter, 250 mm length, 5 μm particle size). At a flow rate of 1 ml/min, the mobile phase consisted of water (A) and acetonitrile containing 0.05% trifluoroacetic acid (B). The mobile phase used a linear gradient program, which was implemented in the following manner: The percentages of grade A are as follows: 82% for 0 min, 80% for 0–5 min, 60% for 5–8 min, 60% for 8–12 min, 82% for 12–15 min, and 82% for 15–16 min. For monitoring, the multi-wavelength detector was adjusted to 280 nm. A 10 µl volume of each solution sample was injected. The temperature of the column was kept constant at 35 °C.

#### Microcapsulation of probiotics

##### Bacterial origin and culture conditions

To initiate each probiotic strain used in this study, 100 ml of MRS broth was used twice^16^. The flasks containing the inoculated samples were incubated at 37 °C for 48 h. Following this, the cultures were transferred into flasks containing 1 L of MRS broth and maintained at the same temperature for an additional 48 h. Subsequently, the bacterial biomasses of each probiotic strain were collected by subjecting them to centrifugation at a speed of 5000 rpm for 15 min at a temperature of 4 °C. Before being used in the feeding experiment, the cell biomasses were kept at 5 ± 2 °C after being suspended in an equivalent amount of MRS broth^4,17^.

##### Viable counts of *Lactobacilli* in MRS suspension

Each strain’s suspended biomass, measuring 1 ml, was separately mixed with 9 ml of sterile physiological saline (0.85% NaCl w/v). The resultant mixture was then diluted in a series of up to 10^− 10^. MRS agar is then poured and allowed to harden after 1 ml of each dilution is plated onto sterilized petri plates in triplicate. 48 h of anaerobic incubation were spent at 37 °C for the plates. The counts were quantified as colony-forming units/ml^18^.

##### Preparation of culture biomass suspension for encapsulation by freeze-drying processing

The *Lactobacillus plantarum* NRC AM10 and *Lactobacillus rhamnosus* NRR B-442 cultures were grown overnight in MRS broth. The bacterial biomass was then collected by centrifuging at 5000 rpm for 15 min at a temperature of 4 °C. The biomass was centrifuged once more while being cleaned with a sterile saline solution (0.85% NaCl) using the same parameters^4^. The obtained biomass was suspended with 10% whey protein as a protective material during freezing and freeze-drying. The bacterial suspension was frozen at a temperature below − 20 °C for 48 h in lab-scale freezers. The frozen bacterial suspension is transferred directly to the Free Zone, a Labconco freeze dryer console, specifically a 12 L stopper tray dryer, in the USA. The bacterial suspension was freeze-dried at -50 °C for 48 h. The method was based on removing the frozen water from the suspension by sublimation. The obtained lyophilized bacterial powder was stored at 4 °C, and 1 g of powder contained approximately 10^11 CFU^4,17^.

##### Determination of encapsulation efficiency of Pb

The determination of encapsulation efficiency (EE) was conducted using the next equation:

$${\text{EE }} = {\text{ Lo}}{{\text{g}}_{{\text{1}}0}}{\text{N}}/{\text{Lo}}{{\text{g}}_{{\text{1}}0}}{\text{N}}0{\text{ }} \times {\text{ 1}}00$$

Here:

N refers to the number of bacterial cells present within the microcapsules following.

N0 represents the number of free bacterial cells introduced to the whey mixture throughout the creation of the biomass suspension^19^.

#### Spirubiotic sachets formulation

Spirubiotic sachets were formulated by adding effective doses of SMs powder (20 g) and Pb powder (1 g = 10^11^CFU) together in the sachet, which would be added to yoghurt, other food, or water and consumed by the patient for 3 months. The dose in the suchet will deliver a dose of Sp (2 g) and Pb (10^11^CFU).

#### Human study design

##### The physic-sensory characterization test

Spirubiotic sachets were tested on 10 volunteers to assess the flavor, color, odor, texture, and taste of the compound. A score range of 0–10 will be assigned to each character.

##### Eligibility criteria

The study was initiated after obtaining permission from the Ethical Committee of the NRC, Egypt. Patients must receive all information about the intervention and provide their informed consent. Written informed consent was obtained before inclusion. Fifty newly diagnosed cases with metabolic syndrome were collected from the Complementary Medicine Clinic, NRCE, NRC, Egypt. Ages eligible for study: the ages were 18 Years and older, documented MeS, and no other health problems. All patients agreed to take the supplements. Exclusion Criteria are advanced chronic liver, renal, or respiratory diseases, or currently using nutritional supplements.

##### Ethical approval

The trail protocol is apart of the project entitled: The use of previously Microencapsulated dietary supplements as health promote which was approved by the Medical Research Ethical Committee (MREC) (NRC, Egypt), Federal Wide Assurance (FWA) (00014747) and RHDIRB: (2017103002) under clinical trail registration number (13050210), the date of decision (06/02/2023) and the renewal date of decision (25/02/2025). All the relevant guidelines and recommendations were followed during the conduct of this research in accordance with the provision of the relevant Egyptian laws, the Egyptian Drug Authority (EDA), Ministry of Health and Population (MOHP) and with Helsinki declaration, good medical and laboratory practice (GCP & GLP) guidlines, and World Health Organization (WHO) rules regarding the scientific research ethics. This study was registered at Universal Trial Number (UTN) (U1111-1325-1939). Moreover, this study is approved by the Pan African Clinical Trial Registry (PACTR) (pactr.samrc.ac.za), the date of the registration is 17/07/2025, and the identification number for the registry is (PACTR202507899407559).

##### Clinical examinations

The presence of at least 3 MeS manifestations was confirmed to diagnose MeS, according to Chen et al.^20^. NCEP ATP111 mentioned at least 3 criteria for diagnosis (BMI ≥ 30, WC 40 inches for males, 35 for females, TG more than 150 mg/dL, HDL less than 40 mg/dL, fasting blood glucose more than 100 mg/dL, and blood pressure 130/85 mmHg systolic/diastolic^21^. The patients who are enrolled received a dose of one *spirubiotic sachet* 21 g/day (20 g SMs equivalent to 2 g Sp and 1 g of lyophilized probiotic (Bp) 10^11^CFU for 3 months.

##### Nutritional assessments

Questioner sheets, dietary habits, and 24 h dietary recall data were collected. The content of macronutrients from protein, fat, CHO, and energy were assessed.

##### Anthropometrice measures

The anthropometric measurements in this investigation tracked changes in body weight, assessed to the nearest 0.1 kg, and height, measured to the nearest 0.5 cm, using a stadiometer (Holtain; Crosswell, Wales) at the beginning and conclusion of the study. Body mass index (BMI) is computed as follows:

$${\text{BMI}} = {\text{weight}/\text{height}^{2}}$$

Moreover, waist and hip circumference measurements were used to determine changes in body mass regionally.

##### Body composition analysis

Preliminary assessments were conducted before treatment to verify the diagnosis, and post-intervention evaluations were performed to assess any resulting changes. Body composition was evaluated using a bipolar body electrical bioimpedance device. The calibrated signal generator (Biodynamics Model 310e, Seattle, WA, USA) produces an electric current with a magnitude of 0.8 mA and a frequency of 50 kHz.

##### Biochemical analysis

Leptin, Insulin, Ghrelin, and Irisin levels were measured using ELISA kits. The enzymatic colorimetric technique^22^ was employed to determine glucose levels. Enzymatic colorimetric methods were used to determine the lipid profiles, which included total cholesterol, HDL, triglycerides, and LDL^23–27^. Colorimetric techniques were used to measure the levels of plasma malondialdehyde (MDA) and plasma catalase activities^28,29^. Plasma alanine aminotransferase (ALT) and alkaline phosphatase (ALP) activity were assessed using colorimetric techniques to determine liver function^30,31^. Urea, creatinine, and uric acid were determined using colorimetric techniques^32–34^. The determination of GGT was performed using a colorimetric approach, as described by Persijn and Van der Slike^35^. Zinc was determined using a colorimetric technique developed by Johnsen and Eliasson^36^. The presence of phosphorus was determined using a colorimetric approach, as described by El-Merzabani et al.^37^.

#### Statistical analysis

The quantitative data analysis was performed using Microsoft Excel and SPSS software, with a primary focus on characterizing the sample and comparing group means. Descriptive statistics were calculated to determine the mean and standard deviation of key variables. To test for statistically significant differences between group means, t-tests were used for comparisons between two groups, and one-way ANOVA was employed for comparisons involving three or more groups. Additionally, simple regression analysis was employed to model and quantify the strength and direction of the relationship between selected independent variables and a dependent outcome variable, enabling prediction and a deeper understanding of the factor’s influence. A significance level of *P* ≤ 0.05 was considered statistically significant.

## Results and discussion

### Characterizations of spirubiotic sachet microcapsules

#### Zeta sizer

The polydispersity index (PDI) and average droplet sizes of nanoemulsions comprising various solutions were evaluated using the Malvern Zetasizer Nano Z instrument. The PDI values serve as a measure of the evenness of droplet size distribution, where lower values suggest a greater level of uniformity^38^. For spirulina microcapsules, the PDI was reported as 0.675 ± 0.01 after the homogenization process, with a droplet diameter of 298.2 ± 3.25 nm. Many parameters, including the emulsion composition, operating conditions, and interaction chamber, may affect the droplet size in nanoemulsions created during homogenization^39^.

The ζ-potential is a quantitative measurement that indicates the degree of electrostatic attraction or repulsion between particles. A substantial ζ-potential in emulsions plays a crucial role in preventing droplet coalescence and aids in maintaining emulsion stability^39^. The ζ-potential measurements of spirulina microcapsules yielded a value of -31.3 ± 0.84 mV, indicating a negative charge on the external surface of the droplets. This negative charge is likely attributed to the presence of carboxylate groups resulting from the addition of whey protein concentrate^40^. Electrostatically stable emulsions are often defined as having ζ-potential values more than + 30 mV or less than − 30 mV^39^. The presence of a negative ζ-potential in this investigation indicates that the emulsion droplets exhibit strong stability^41^.

#### Surface morphology of SMs

**Fig. 1:**
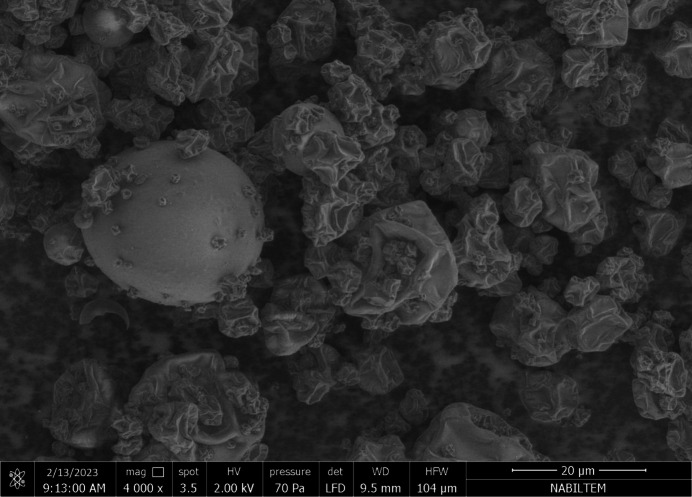
Surface morphology of spirulina microcapsules.

The scanning electron microscopy (SEM) technique was used to study the microstructure of the SMs. Folds were evident on the microcapsules’ surface, as shown in Figure 1, a frequent occurrence in powders made by spray drying^42^. The formation of folds is a result of rapid shell formation during the early stages of drying and is influenced by the system’s composition. The addition of whey protein concentrate in this study contributes to the activity on the microcapsule surface and the development of folds, resulting in a smoother surface appearance. During drying, the protein-rich film on the droplet surface exhibits elasticity, allowing for expansion and subsequent shrinkage of the particle^43^. The results align with the observations presented by Bhusari et al.^44^, who found that whey protein concentrate led to an increase in particle shrinkage.This may be attributed to the rise in the protein content of the carrier agents.

#### TPC and TFC

The total flavonoid content (TFC) of 1.20 ± 0.05 mg of rutin and the total phenolic content (TPC) of 5.17 ± 0.03 mg of catechin/g were found in the SMs extract, according to the results. The content of phenolic and flavonoid compounds strengthens the ability of Sp to act as an antioxidant and weight-controlling agent^45^.

####  The encapsulation efficiency (EE)

EE was determined to be 91.3 ± 3.25%. EE is a crucial criterion for the proper encapsulation of the core material. The reduced phenolic content on the surface of spirulina microcapsules signifies a more effective encapsulation process. Elevated EE guarantees the delivery of active substances to target organs without change^46^.

#### Antioxidant capacity of SMs

Results showed high antioxidant activity. DPPT activity was 0.32 ± 0.01 µmol Trolox/g. The ability of Sp to donate hydrogen ions and hence decrease oxidant stress is a mechanism that targets and blocks the precipitation of many diseases. Free radicals have the potential to harm many cells and organs, leading to the development of various disorders^47^.

#### HPLC of phenolic extract (PE)

**Fig. 2:**
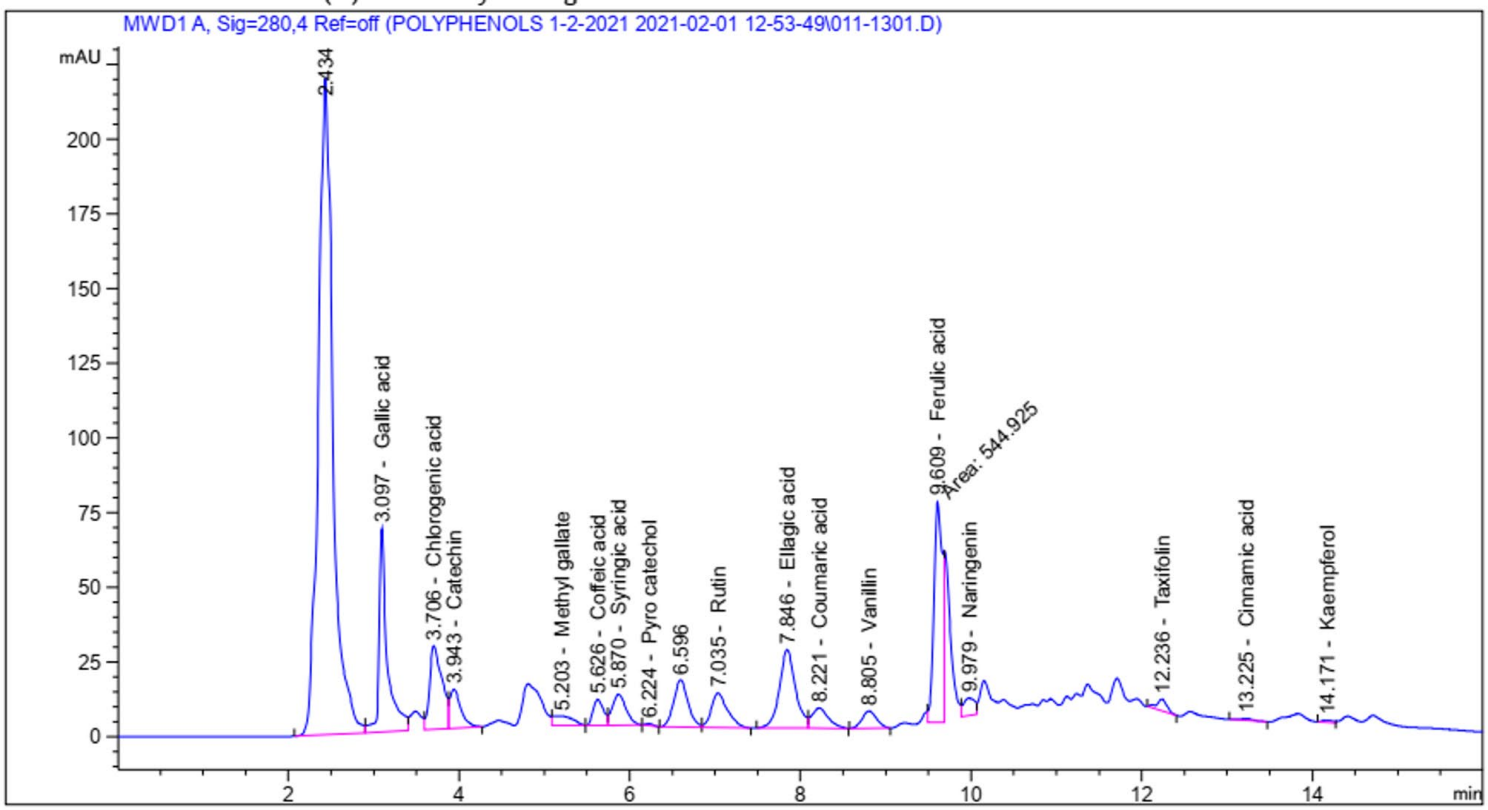
HPLC profile of spirulina phenolic extract.

The HPLC profiles of different SM fractions were analyzed to determine the presence and quantities of 16 phenolic compounds. Each fraction’s discovered phenolic compounds are shown in Table 1, while Figure 2 illustrates the peaks observed at various retention times. The analyzed SM was evaluated for the presence and concentrations of specific phenolic compounds. Results revealed the concentrations of flavonoids such as Rutin (1202.56 µg/g), Naringenin (1102.61 µg/g), Catechin (773.53 µg/g), (113.80 µg/g), Vanillin (90.81 µg/g), Methyl gallate (37.60 µg/g), Pyrocatechol (15.06 µg/g), Taxifolin, and Kaempferol (12.38 µg/g). Some flavonoids, especially Rutin and Kaempferol, have been shown to have anti-obesity effects. They may help reduce body weight, inhibit the accumulation of fat cells, and increase fat metabolism. Flavonoids are known to exert their mode of action by scavenging or chelating mechanisms, as stated by Cherrak et al.^48^. Spirulina exhibits a remarkable capacity for synthesizing phenolic and flavonoid compounds, exhibiting a superior yield compared to conventional plant-derived sources. Polyphenols are powerful antioxidants because of their hydroxyl groups’ ability to release hydrogen and their ability to donate electrons to prevent the production of free radicals produced by oxidative stressors, which consequently causes metabolic disruptions in meS^47^. These antioxidant effects can potentially contribute to weight management and the prevention of obesity^3,49^. Furthermore, phenolic acids belonging to the subclass of hydroxybenzoic acids were analyzed. Results showed Syringic acid (326.52 µg/g), Gallic acid (2047.38 µg/g), Ellagic acid (1412.36 µg/g), and., additionally, hydroxycinnamic acids such as Coffeic acid (158.69 µg/g), Chlorogenic acid (1261.06 µg/g), Ferulic acid (1102.61 µg/g), and Cinnamic acid (98.97 µg/g). The phenolic acids, especially Gallic acid, Ellagic acid, Chlorogenic acid, and Ferulic acid, have been shown to have anti-obesity effects. They may help regulate adipocyte differentiation, inhibit fat cell growth, and promote fat metabolism. These effects can potentially contribute to weight management and the prevention of obesity, which is a key component of metabolic syndrome^50^.

**Table 1:** HPLC analysis of spirulina phenolic extract.

Phenolics	Area	Conc.(µg/g)
Gallic acid	452.29	2047.38
Chlorogenic acid	284.04	1261.06
Catechin	116.29	773.53
Methyl gallate	51.25	37.60
Coffeic acid	73.20	158.69
Syringic acid	115.63	326.52
Pyro catechol	4.12	15.06
Rutin	151.39	1202.56
Ellagic acid	344.24	1412.36
Coumaric acid	90.44	98.97
Vanillin	67.85	90.81
Ferulic acid	544.93	1102.61
Naringenin	53.73	181.05
Taxifolin	32.51	113.80
Cinnamic acid	7.24	4.30
Kaempferol	5.90	12.38

### Freeze dried probiotic

Table 2 illustrates the counts of mixed probiotic strains in MRS + 10% whey protein and in biomass suspension + 10% whey protein. Freezing injuries can decrease the viability of bacterial cells due to the formation of intracellular ice crystals. The addition of dairy proteins, such as skim milk or whey protein, serves as a protective agent, improving the stability and survival of cells after freezing. The obtained data showed that the encapsulation efficiencies recorded were 94.43% and 92.36% in biomass suspension + 10% whey protein and MRS + 10% whey protein, respectively. The results align with those presented by Mabrouk et al.^17^, who reported that encapsulating probiotics with milk proteins can maintain cell structure and improve probiotic viability in simulated gastric conditions and soft cheese.

Probiotic bacteria (*Pb*) have numerous health benefits, including anti-hyperlipidemic and anti-hyperglycemic effects^3,4^. It has been demonstrated that it decreases the activity of mutagenic enzymes^5^, leading to lower levels of lipids and sugars in individuals with type 2 diabetes and liver steatosis. Additionally, it has been seen to alleviate metabolic syndrome in rats^3,4^.

**Table 2:** The results of counts of probiotic after freeze-drying and E.E.

Treatments	Counts CFU/g	Counts log CFU/g	Encapsulation efficiency EE
Initial count in MRS (N0)	25 × 10^9^	11.39	-
The count after freeze-drying in MRS + 10% Whey protein (N)	33 × 10^8^	10.52	92.36
The count after freeze-drying in biomass suspension + 10% Whey protein (N)	57 × 10^8^	10.76	94.43

### Human study

#### The physico-sensory test

Results showed that the spirubiotic sachet product is palatable, with scores of 80–90% for taste, color, texture, flavor, and odor.

#### Eligability results

Fifty diagnosed cases with metabolic syndrome were collected from the Complementary Medicine Clinic, NRCE, NRC, Egypt. They were presented with their previous biochemical results that confirmed the diagnosis of MeS while respecting the exclusion criteria. The biochemical parameters used to make the enrollment decision for all cases were as follows: random glucose (148 ± 4.8), TC (245 ± 17.5), HDL (38.5 ± 2), LDL (153 ± 6), and TG (141 ± 7). Some cases that started the treatment did not continue the study, and some were not committed to the treatment regimen. Only 30 patients completed the study (Due to the coronavirus pandemic).

#### Nutrtional status of patients

The composition of food consumed in a 24-hour dietary recall was analyzed before and after treatment. Tables 3 and 4 demonstrated the different macronutrients taken by the patients before and after treatment, respectively. They demonstrate the main macronutrients (MN): protein (PR) (source of muscles built), fat, carbohydrates (CHO), and energy (source of power). The metabolic functioning of various organs is known to be influenced by diet habits; long-term unbalanced nutritional habits can contribute to many diseases, such as hypertension, hyperglycemia, obesity, and MeS^51^.

The calories consumed by the patient before and after the trial significantly declined (2622 ± 436 to 1932 ± 307 calories/day, respectively), as patients were assigned to a specific diet regimen. Patients reported and observed cuts in their sugar intake, which resulted in feelings of hunger. This was also followed in previous studies by Zeinalian et al.^52^.

Analysis of food composition before and after treatment in a 24-hour dietary recall (one-day diet) revealed a significant increase in protein intake from chicken, fish, bread, dairy products, and cereals in the after-treatment group. As consumption was increased from these food groups, significant increases in fat, CHO, and energy groups were noted. Protein intake was higher in the after group compared to the before-treatment group (ranging from 0.3 to 2.6 g/kg BW/d compared to 1.0 to 3.3 g/kg BW/d) in the before and after groups, respectively, and categorizing the after group as high-protein groups. In this study, protein intake was compared to the Dietary Reference Intakes (DRI). Specifically, the proportion of participants consuming less protein than the estimated average requirement (EAR) was examined. The EAR is 50 g/day for men and 40 g/day for women.

Additionally, protein intake was compared to the reference nutrient intake (RNI), which is 60 g/day for men and 50 g/day for women. Furthermore, protein intake was also compared to the protein EAR/kg body weight (0.73 g/kg/day) and the protein RNI/kg body weight (0.91 g/kg/day)^53^. The post-group exhibited a greater consumption of protein compared to the preceding sources. In 2018, the Korean Geriatric Society and the Korean Nutrition Society suggested increasing the recommended protein intake to 1.2 g/kg/day, which is 31.4% more than the existing amount^54^. A diet rich in protein and hence high in amino acid intake promotes muscle growth, mitigates the impact of age-related muscular strength decline, and facilitates weight reduction in individuals with obesity^55^. A cross-sectional research of both US Americans and individuals in China found a positive correlation between muscle mass and the consumption of total protein and animal-based proteins^56,57^. The treatment group showed significantly higher protein intake (Table 5). It has been found that dietary macronutrient ratios (protein, carbohydrate, and fat) influence aging and cardiometabolic health more significantly than calorie consumption^58^.

The after-treatment group showed significantly lower CHO% intake compared to the before-treatment group. The percent of energy intake (En%) from CHO in the current study ranged between 12% and 40% in both groups, which is consistent with the recommended percent. The concept of an optimal energy intake to maintain a healthy weight has led to the consideration of total carbohydrates, free sugars, starch, sugars inside the cellular structure of food, and milk sugars. It is recommended that the typical population’s consumption of total carbohydrates be maintained at the dietary reference value of approximately 50% of total dietary energy^59^.

**Fig. 3:**
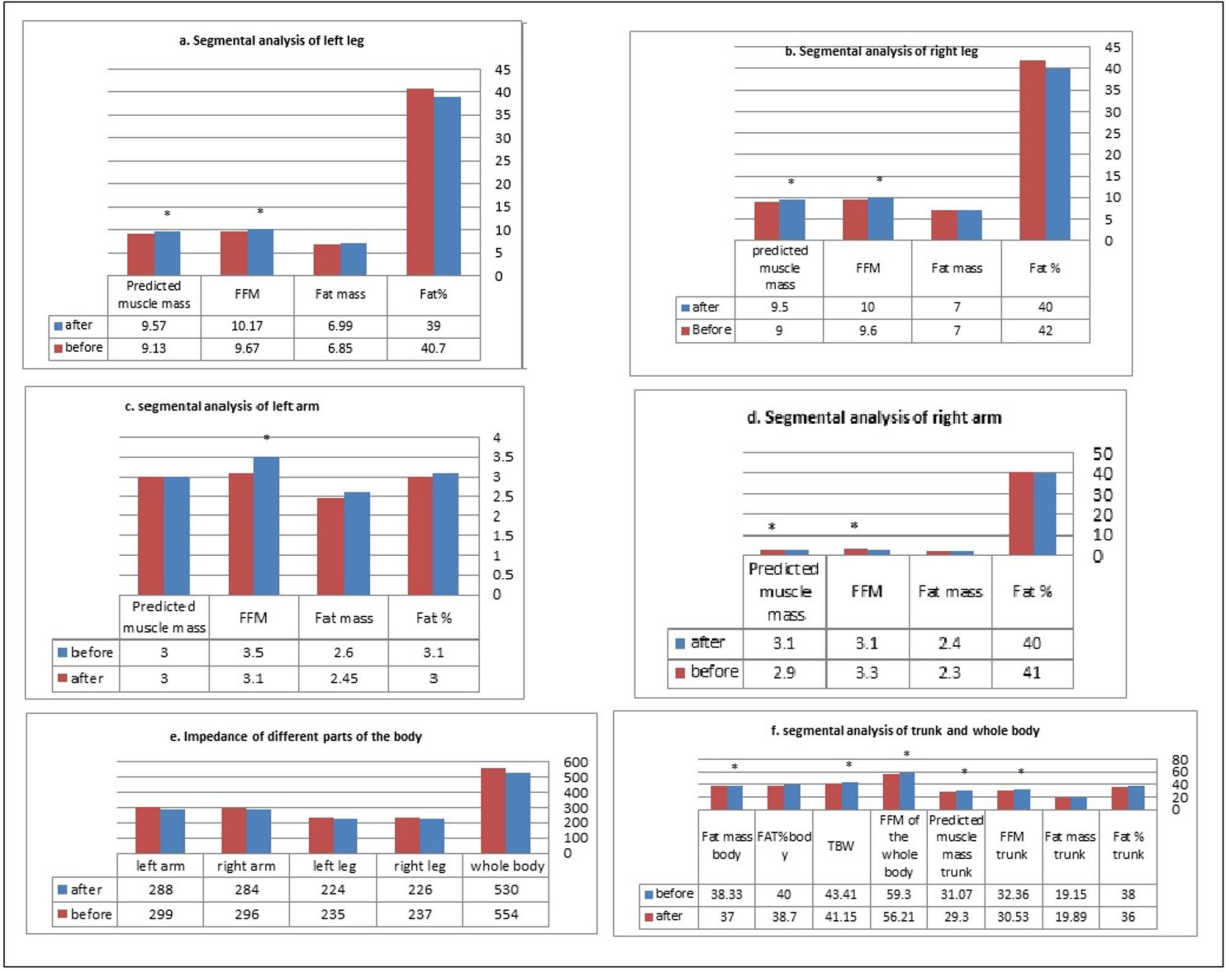
Body composition analysis of all parts of body before and after treatment.

Moreover, Tables 5 and 6 summarizes the 24 h dietary recall. It demonstrates the overall amounts of the three macronutrients (protein, fat, and carbohydrates) represented in g/kg of body weight and their percentage from total intake, as well as total daily energy intake for all patients enrolled before and after treatment. The significant elevation of protein intake and reduction in fat intake and energy were consistent with the increased predicted muscle mass and the reduced fat mass and fat in body composition analysis (Table 7; Figure 3). Anthropometric measures were significantly decreased in the after group affected by this change in food composition (Table 6). The reduction in fat intake in the after group was accompanied by a significant decrease in lipid profile, random blood glucose, and other biochemical parameters (Table 8).

**Table 3:** Intake of macronutrients from food groups (before treatments-MeS).

Diet composition	Macronutrients										
	Dairy	Fish	Chicken	Meat	Cereals	Sweets	Drinks	Oil	Fruits	Vegetables	Bread
Protein (g/d)	4.85 ± 0.37^b^	24.6 ± 0.0^d^	28.29 ± 0.98^e^	13.03 ± 2.07^c^	5.05 ± 0.22^b^	6.55 ± 0.28^b^	0.14 ± 0.03^a^	–	0.49 ± 0.03^a^	1.47 ± 0.33^a^	11.91 ± 1.91^c^
Fat (g/d)	6.005 ± 0.46^bc^	18.5 ± 0^ef^	20.30 ± 0.40^f^	7.53 ± 1.09^c^	4.44 ± 0.12^bc^	16.33 ± 1.04^de^	0.46 ± 0.07^a^	–	0.12 ± 0.003^a^	2.79 ± 0.72^ab^	13.26 ± 3.86^d^
Carbohydrate (g/d)	2.89 ± 0.44^a^	0.40 ± 0.0^a^	3.09 ± 0.26^a^	15.93 ± 1.31^b^	44.49 ± 1.04^c^	15.28 ± 1.22^b^	3.28 ± 0.64^a^	–	5.47 ± 0.14^a^	4.27 ± 0.87^a^	81.68 ± 9.28^d^
Energy (kcal/d)	94.49 ± 5.63^c^	267.0 ± 0.0^e^	341.63 ± 8.42^f^	147.50 ± 16.43^d^	239.12 ± 5.83^e^	320.53 ± 13.02^f^	7.68 ± 0.05^a^	–	38.37 ± 1.72^a^	65.15 ± 3.46^bc^	329.1 ± 42.79^f^

The data are expressed as the mean ± SD. Identical letters in each raw denote that there is no statistically significant difference between the varieties, while different letters indicate a significant difference at a *P* ≤ 0.05 level.

**Table 4:** Intake of macronutrients from food groups (after treatment-MeS).

Diet composition	Macronutrients										
	Dairy	Fish	Chicken	Meat	Cereals	Sweets	Drinks	Oil	Fruits	Vegetables	Bread
Protein(g/day)	9.1 ± 1.2^c^	23.7 ± 0.02^e^	35.85 ± 1.80^f^	11.67 ± 0.37^d^	4.07 ± 0.18^b^	1.72 ± 0.19^a^	0.6 ± 0.00^a^	0.003 ± 0.00^a^	0.51 ± 0.29^a^	0.64 ± 0.12^a^	11.57 ± 0.41^d^
Fat(g/day)	17.52 ± 5.33d	11.23 ± 0.71^c^	37.38 ± 3.11^e^	9.48 ± 0.19^bc^	0.35 ± 0.01^a^	9.10 ± 0.16^bc^	–	4.90 ± 0.06^ab^	0.33 ± 0.42^a^	0.44 ± 0.33^a^	4.61 ± 0.16a^b^
Carbohydrate (g/day)	2.97 ± 0.19^ab^	6.50 ± 0.00^bc^	7.20 ± 0.5^8c^	6.13 ± 1.35^bc^	44.87 ± 2.06^e^	20.84 ± 1.29^d^	9.40 ± 0.00^c^	0.20 ± 0.00^a^	8.70 ± 0.47^c^	3.01 ± 0.87^ab^	81.62 ± 2.92^f^
Energy(kcal/day)	106.36 ± 2.73^b^	165.10 ± 0.28^c^	430.69 ± 21.46^f^	128.65 ± 0.45^b^	236 ± 6.94^d^	132.73 ± 8.39^b^	45.00 ± .00^a^	44.43 ± 0.56^a^	47.20 ± 0.71^a^	22.04 ± 4.65^a^	364 ± 13.04^e^

The data are expressed as the mean ± SD. Identical letters in each raw denote that there is no statistically significant difference between the varieties, while different letters indicate a significant difference at a *P* ≤ 0.05 level.

**Table 5:** Composition of patient’s diet before and after treatment (24 h dietary recall).

Diet composition	Treatment			
	Before treatment-MeS	Before treatment (%)	After treatment-MeS	After treatment (%)
Protein (g/day)	9.04 ± 0.78^ab^	4.72	9.6 ± 0.71^a^	8.98
Fat (g/day)	9.57 ± 0.99^ab^	6.71	8.97 ± 0. 6^a^	5.7
Carbohydrate (g/day)	17.7 ± 1.95^ab^	9.07	17.39 ± 1.67^a^	7.94
Energy (cal/day)	185.1 ± 9.75^ab^	79.5	156.56 ± 9.37^a^	77.38

The data are expressed as the mean ± SD. Identical letters in each raw denote that there is no statistically significant difference between the varieties, while different letters indicate a significant difference at a *P* ≤ 0.05 level.

#### Anthropometric measurement

The pretreatment group was committed to enrollment and the MeS criteria. Results represented in Table 6 showed that all anthropometrics results showed obesity with a BMI ≥ 30 in pretreatment. After treatment, patients’ measures showed a significant decrease in weight, BMI, WC, and HC.

**Table 6:** Anthropometric measurement before and after intervention.

Item	Before treatment-MeS	After treatment-MeS	*P* value*
Weight (kg)	96.78 ± 18.0^*^	94.68 ± 15.4	≤ 0.01
BMI kg/(height)^2^	36 ± 6.9^*^	34.7 ± 6.17	≤ 0.01
WC (cm)	113.5 ± 13*	109 ± 12.3	≤ 0.01
HC (cm)	120.5 ± 8.84^*^	119 ± 39.3	≤ 0.05

The data are expressed as the mean ± SD. A significant difference at a *P* ≤ 0.01 and *P* ≤ 0.05 level. WC (Waist circumference), HC (Hip circumference).

#### Body composition analysis

Table 7 and Figure 3a–f demonstrated the result of body composition analysis (in the body) parameters showing significant improvement in BMI, obesity parameters, and body composition of patients after treatment.

Obesity is linked to several health issues and is seen as a serious public health concern. In the current study, we assessed the effects of microencapsulated spirulina and probiotics (in one spirubiotic sachet) on controlling obesity and related metabolic derangements, as documented by improvements in anthropometric measurements and parameters of body composition analysis. Results showed a significant reduction in body weight, BMI, WC, and HC (Table 6). Body composition analysis revealed a decrease in body fat mass, FFM in all body regions, and total body water, accompanied by elevated predicted muscle mass (Fig. 3). These results align with those obtained in a systematic review and meta-analysis by Zarezadeh et al.^60^.

According to Hernández-Lepe et al.^61^, Sp may help overweight or obese individuals lose weight both with and without a structured physical activity regimen. A study observed that the length of the intervention was longer in trials with lower dosages and that the provided dosage of ≤ 2 g/day had a stronger impact on weight reduction compared with > 2 g/day Sp^60^. The length of the intervention appears to have a greater effect on weight reduction than the dose used. The dosage employed in the present trial, which addressed both goals, was 2 g of spirulina/day for three months, targeting both aims.

The study showed a significant decrease in BW and BMI median (MD) (1, 9 kg, 1.3, respectively). In contrast, WC and HC were decreased (Median of 4.5 cm and 1.5 cm, respectively); these weight losses were higher than those reported in one matched meta-analysis study^62^. A further meta-analysis revealed a substantial decrease in both BW and WC. Nevertheless, no obvious effect on BMI was seen^60^.

Given the nutritional nature of Sp supplementation, it may be used in a paleolithic diet prescription to amplify its positive benefits in those with obesity, potentially. Sp may be included in a low-advanced glycation end-product diet, which has beneficial effects on indicators of glucose and lipid metabolism, especially for those with type 2 diabetes. Hence, it is important to use Sp as a supplementary therapy for those suffering from diabetes mellitus or obesity to avoid and effectively manage concurrent medical conditions^9^.

Probiotics, as a component of the spirubiotic sachet, are considered health promoters. One meta-analysis study agreed with the current results and concluded that probiotic administration was associated with a substantial decline in body weight and fat percentage as opposed to placebo^63^; nevertheless, in another research study that evaluated the efficacy of probiotics in lowering body weight in patients with related metabolic diseases who were overweight or obese, the effect on fat mass was not statistically significant, results stated that a significant BMI, WC, and HC reduction were observed on the contrary, there was no significant decrease in body weight^6,64^. Substantial cuts in BMI, BW, WC, waist-to-height ratio, and blood glucose were documented after supplementation with probiotics compared to a placebo^65^. Others disagreed with these results, mentioning no change or a minimal effect on anthropometric measures^66^.

The positive impacts of probiotics were more noticeable in studies lasting 12 weeks or longer, with a daily probiotic dosage of at least 10^− 10^ CFU, in the form of sachets, pills, or powder. Overall, a recent meta-analysis has found that incorporating various strains of probiotics into one’s diet or taking them in capsule form can result in a notable decrease in body fat percentage and weight. However, it is important to note that the impact of probiotics on body weight appears to vary depending on the specific strain used, and further research is needed to fully understand the mechanisms by which certain microorganisms contribute to weight reduction^65^.

Microencapsulation of Sp and Pb in the current study, as well as their combination in a single sachet, was an innovative idea to deliver the active components of the sachet to target organs protected by whey protein and gum Arabic, similar to plastic microspheres, which protect against digestion and external derangement effects. The Spirubiotic sachet contains both microencapsulated Sp and an augmented spirubiotic sachet effect, which showed a higher reduction in body measures and yielded results higher than those previously mentioned in studies.

**Table 7:** Body composition analysis before and after intervention.

Item	Before treatment-MeS	After treatment-MeS	*P* value*
BMR	7561.4 ± 12	7137.1 ± 834.9	≤ 0.05
FAT%	40 ± 7	38.7 ± 9	NS
Fat mass	38.33 ± 14.32	37.±13.7	≤ 0.05
Impedance of whole body	530.4 ± 84.68	554.3 ± 78.7	NS
Impedance of right leg	225.6 ± 32.2	236.5 ± 26.9	NS
Impedance of left leg	223.8 ± 32.9	235.2 ± 25.6	NS
Impedance of right arm	283.6 ± 48.84	296.2 ± 49.5	NS
Impedance of left arm	288.2 ± 56.35	299 ± 54.14	NS
Segmental analysis of right leg			
Fat %	0.4048 ± 0.114	0.415 ± 0.086	NS
Fat mass	7.26 ± 2.93	7.04 ± 2.31	NS
FFM	10.14 ± 1.83	9.59 ± 1.46	≤ 0.05
Predicted muscle mass	9.54 ± 1.67	9.0 ± 1.35	≤ 0.05
Segmental analysis of left leg			
Fat%	0.39 ± 0.12	0.407 ± 0.09	Ns
Fat mass	6.99 ± 2.98	6.85 ± 2.34	Ns
FFM	10.17 ± 1.93	9.67 ± 1.62	≤ 0.05
Predicted muscle mass	9.57 ± 1.74	9.13 ± 1.54	≤ 0.05
Segmental analysis of right arm			
Fat %	0.40 ± 0.13	0.41 ± 0.12	NS
Fat mass	2.411 ± 1.29	2.271 ± 1.03	NS
FFM	3.3 ± 0.79	3.12 ± 0.76	≤ 0.05
Predicted muscle mass	3.1 ± 0.76	2.92 ± 0.73	≤ 0.05
Segmental analysis of left arm			
Fat %	3.074 ± 8.37	3.0 ± 8.14	NS
Fat mass	2.58 ± 1.4	2.451 ± 1.18	NS
FFM	3.42 ± 0.86	3.15 ± 0.74	≤ 0.05
Predicted muscle mass	3.21 ± 0.82	3 ± 0.67	≤ 0.05
Segmental analysis of the trunk			
Fat %	0.38 ± 0.07	0.36 ± 0.06	NS
Fat mass of the trunk	19.15 ± 6.10	19.89 ± 6.08	NS
FFM of the trunk	32.36 ± 5.37	30.53 ± 4.08	≤ 0.05
Predicted muscle mass of the trunk	31.07 ± 5.28	29.30 ± 4.02	≤ 0.05
FFM of the whole body	59.3 ± 10.44	56.21 ± 8.56	≤ 0.05
TBW	43.41 ± 7.63	41.15 ± 6.26	≤ 0.05

The data are expressed as the mean ± SD. A significant difference at a *P* < 0.05 level.

#### Biochemical parameters

Metabolic syndrome (MeS) is characterized by the presence of abdominal obesity, elevated triglyceride levels, low levels of high-density lipoprotein cholesterol (HDL-C), increased blood pressure, and higher fasting glucose levels^3,4^. Spirulina, a cyanobacterium of considerable value, can serve as a nutritional supplement to manage metabolic syndrome and its complications, and act as a prebiotic that enhances probiotic modulation^5,7^. Microencapsulation will protect symbiotics, allowing them to target their functions^8^. Individuals diagnosed with Metabolic Syndrome (MetS) typically exhibit an aberrant lipid profile, characterized by elevated triglyceride (TG) and low-density lipoprotein (LDL) levels. These lipid abnormalities serve as precipitating factors for the appearance of several issues, including atherosclerosis, cardiovascular disease (CVD), fatty liver, and stroke^67,68^. The presence of elevated non-esterified free fatty acids in the bloodstream, resulting from hyperlipidemia and increased triglyceride levels, contributes to the accumulation of fat in both organ cells and the walls of blood vessels. These effects were observed in the pre-group, which exhibited higher levels of serum LDL, lower levels of HDL, and elevated triglycerides compared to the post-control group.

The findings of this study indicate that administering spirulina and probiotic bacteria microcapsules as a post-treatment intervention resulted in a significant reduction in total cholesterol (TC), low-density lipoprotein (LDL), and triglyceride (TG) levels. These observed changes have the potential to have a crucial function in the prevention of dyslipidemias^68,69^. The ingestion of spirulina has been observed to mitigate lipid accumulation within the liver through the mechanism of diminishing the infiltration of macrophages into visceral adipose tissue^70^. The presence of phenylalanine in the substance may also lead to an elevation in the secretion of cholecystokinin, thereby potentially exerting an inhibitory effect on appetite^71^. Spirulina is known to possess antioxidants that are significant in the context of weight management^72,73^ and have the potential to enhance energy expenditure and impede the processes of adipocyte differentiation and lipase activities^74^. Hence, it is anticipated that incorporating spirulina supplementation would yield enhancements in the weight loss process^75^. However, to provide a clear explanation of the specific process, additional research is needed to determine how spirulina can potentially aid in weight loss. Nevertheless, the findings from the current study and the previous study on an animal model of Metabolic Syndrome (MeS) align with the results of a recent study that assessed the protective effects of spirulina against diet-induced obesity and metabolic disorders. Further studies have shown that over 12 weeks of consuming spirulina supplements, there was a notable decrease in the increase of body weight, as well as in levels of plasma glucose, insulin, and triglycerides^3,76^.

Insulin plays a pivotal role in maintaining glucose homeostasis and facilitating enhanced uptake of glucose in peripheral tissues. A study found that the pretreatment group, which received supplementation of spirulina and probiotic bacteria microcapsules, exhibited higher blood glucose levels and lower insulin levels compared to the post-group^9^. Spirulina has the potential to exhibit insulin-like properties, such as stimulating the B cells of Langerhans to enhance insulin synthesis, improve insulin sensitivity at receptor sites, and reduce serum glucose levels. Furthermore, the fiber content, protein composition, and bioactive polypeptides generated during digestion could potentially contribute to decreased glucose absorption^73,76^.

In the current study, significantly elevated levels of leptin and irisin were observed in the pretreatment-MeS patients in comparison to those in the after-treatment group. Leptin and Irisin levels have been proposed as potential biomarkers for predicting the presence of metabolic syndrome, abnormal glucose metabolism, insulin resistance, fat mass, and the risk of developing cardiovascular disease^77^. The concentration of serum leptin has a positive correlation with the amount of total body fat, thereby transmitting a signal to the brain and hypothalamus to regulate dietary intake and metabolic rate^4^. Elevated levels of irisin in the bloodstream have been found to have a positive impact on obesity and glucose imbalance, as they are closely associated with increased energy expenditure. In the current study, the improvement in obesity parameters, including total body fat and body composition values (Tables 6 and 7), in the after-treatment group was reflected in the decreased levels of leptin and irisin. This observation is consistent with previous research that has reported elevated levels of leptin in individuals with metabolic syndrome and obesity^4^.

Ghrelin is known for its ability to enhance food consumption and promote weight gain due to its orexigenic, adipogenic, and somatotrophic properties^41^. The concentration of ghrelin in humans is observed to be reduced in conditions characterized by obesity and increased in anorexia nervosa^78^. In the current study, the observed decrease in anthropometric measures after treatment in Table 6 was reflected in the level of ghrelin, which was elevated. Similar observation showed a significant inverse relationship between basal ghrelin levels and BMI, waist-to-hip ratio, and waist circumference^79^. There is a positive correlation between reduced ghrelin concentrations and an increased prevalence and severity of metabolic syndrome^80^. The primary factor contributing to this phenomenon can be attributed to adiposity, which plays a significant role in influencing various other characteristics associated with metabolic syndrome (MeS)^81^.

The levels of catalase, MDA, phosphorus, and zinc exhibited a notable increase in the post-treatment group compared to the pretreatment group. This decrease may be ascribed to the administration of spirulina and probiotic bacteria microcapsules, which effectively regulate oxidative stress^47,50,82^. The generation of oxygen radicals resulting from the inflammatory process and lipid peroxidation led to a reduction in antioxidant levels and zinc availability, due to the heightened requirement for combating oxidative stress. This phenomenon was observed in the before-treatment group compared to the after-treatment group. Oxidative stress has been documented in prior studies on metabolic syndrome MeS^47,50,83^ (Table 8).

The pre-group exhibited elevated levels of serum liver γ-GT, which subsequently decreased to levels considered within the normal range in the post-group. The distinctive impact exerted by spirulina and probiotic bacteria microcapsules on liver function can explain this effect. γ-GT is a transmembrane protein that plays a role in the extracellular degradation of glutathione (GSH) mainly in the liver^84^. Serum γ-GT levels that are elevated have been proven to be suggestive of the onset of metabolic syndrome (MeS)^85^. The increase in γ-GT levels is closely linked to hepatic steatosis, which is itself strongly correlated with and serves as the sole hepatic biomarker of MeS. Additionally, there is an association between increased levels of serum γ-GT and the presence of MeS-related hypertension, as well as an elevated susceptibility to the development of diabetes^4^.

The present study demonstrated that the levels of urea and creatinine in both the pre- and post-groups were within normal ranges, suggesting that the utilization of spirulina and probiotic bacteria microcapsules did not pose any significant safety concerns. An increased level of uric acid serves as a robust indicator for the initiation of metabolic syndrome, and its elevation demonstrates a significant association with various cardiovascular risk factors. The biomarker in question appears to possess the highest degree of reliability in discerning individuals who exhibit metabolic syndrome^86,87^.

**Table 8:** Biochemical parameters of different groups.

Parameters	Groups		
	Before treatment-MeS	After treatment-MeS	*P*‑value
Glucose (mg/dL)	148.4 ± 5	111.7 ± 3.7	˂0.05
γ-GT (U/L)	36.2 ± 5.5	27 ± 5.9	˂0.05
Leptin (ng/ml)	24.8 ± 2.2	16.5 ± 1.3	˂0.05
Insulin (µU/ml)	9.4 ± 0.8	15.3 ± 0.7	˂0.05
Ghrelin (pg/ml)	326.8 ± 10.7	681.6 ± 11.1	˂0.05
Irisin (ng/ml)	247.5 ± 2.8	172.5 ± 6.7	˂0.05
Catalase (mg/dL)	52.4 ± 5.2	68.3 ± 6.2	˂0.05
MDA (mg/dL)	6.8 ± 0.6	10.2 ± 1.1	˂0.05
Total cholesterol (mg/dL)	233.9 ± 18.7	144 ± 12.8	˂0.05
HDL (mg/dL)	49.1 ± 2.1	65.1 ± 5.1	˂0.05
LDL (mg/dL)	153.7 ± 5	71 ± 12.4	˂0.05
Triglycerides (mg/dL)	127 ± 8.5	91.8 ± 6	˂0.05
ALT (U/L)	37.3 ± 2.1	25.7 ± 3	˂0.05
ALP (U/L)	77.1 ± 2.4	41.4 ± 4.6	˂0.05
Creatinine (mg/dL)	0.7 ± 0.1	0.8 ± 0.2	0.63
Urea (mg/dL)	33.6 ± 3.1	33.6 ± 4.1	0.495
Uric Acid (mg/ dL)	5.3 ± 0.3	4.1 ± 0.5	˂0.05
Phosphorus (mg/dL)	9.7 ± 1.4	12 ± 1.2	˂0.05
Zinc (µg/dL)	122.4 ± 4.9	181.3 ± 8	˂0.05

The data are expressed as the mean ± SD. A significant difference at a *P* < 0.05 level.

## The significance and limitations

The research on the spirubiotic sachet (microencapsulated *Spirulina platensis* (Sp) and probiotic bacteria (Pb)) is significant because it introduces an innovative, potentially more effective approach to managing Metabolic Syndrome (MeS) by leveraging the synergistic beneficial effects of Sp and Pb in a single, convenient, microencapsulated delivery system. The study’s findings suggest the sachet, along with a tailored diet, can effectively reverse a majority of metabolic abnormalities—including improvements in hyperlipidemia, weight gain, and hyperglycemia—by reducing adverse changes in anthropometric, body composition, and biochemical parameters. However, a key limitation is the small sample size (thirty diagnosed MeS cases), which may limit the generalizability of the results. Furthermore, the reliance on a concurrent tailored diet regimen makes it difficult to definitively isolate the precise extent of the spirubiotic sachet’s impact versus the dietary changes, as both factors contributed to the reported positive outcomes over the three-month intervention period.

## Conclusions and recommendations

Metabolic Syndrome (MeS) is recognized as a major and pervasive global health challenge, necessitating intensive research. This study successfully contributed to the development of the spirubiotic sachet, a novel food supplement containing active functional Sp + Pb compounds that are known to influence MeS components positively. To ensure optimal efficacy and targeted delivery to the digestive tract, these beneficial substances were protected through a process of microencapsulation. Drawing on previous animal model testing, the active compounds demonstrated promising effects on improving MeS indicators to varying degrees, leading to the evaluation of the product in human MeS patients. The findings support the hypothesis that food supplements like the spirubiotic sachet can effectively aid routine diet and drug regimens, providing a synergistic tool to enhance MeS control and potentially shorten the time required to achieve beneficial results, thereby strongly recommending the execution of further studies to assess and validate these supplements fully.

## Data Availability

The data analyzed during the current study available from the corresponding author on reasonable request.
